# Bringing fruity meat dishes of Ottoman cuisine into businesses

**DOI:** 10.1186/s42779-021-00107-2

**Published:** 2021-10-20

**Authors:** Osman Güldemir, Onur Tugay, Gökhan Şallı, Emrah Yıldız, Seher Çelik Yeşil

**Affiliations:** 1 Department of Cookery, Vocational School of Eskisehir, Anadolu University, Eskisehir, 26210 Turkey; 2 Scientific Research Projects, Anadolu University, Eskisehir, 26210 Turkey; 3 Department of Gastronomy and Culinary Arts, Faculty of Tourism, Anadolu University, Eskisehir, 26210 Turkey; 4 Department of Tourism Management, The Graduate School of Social Sciences, Kastamonu University, Kastamonu, 37150 Turkey

**Keywords:** Traditional cuisine, Ethnic foods, Ottoman cuisine, Culinary education, Fruity meat dishes

## Abstract

Aim of this research is to promote the inclusion of traditional Ottoman fruity meat dishes into business. Chefs working in various food establishments in Eskisehir were given training on fruity meat dishes for a total of 40 h in 10 training segments. Interviews were conducted, analyzed and evaluated in terms of the themes. The inclusion of fruity meat dishes of Ottoman cuisine in businesses would pose no difficulty at any stage of preliminary preparation, cooking, or budgeting. The inclusion of these dishes in the fast-breaking menu during the Ramadan and making small adjustments in accordance with customer feedback would help include these meals. During this study, the kitchen staff gained insight regarding Ottoman cuisine, they became more adept at implementing fruity meat techniques, and the business managers and owners showed much care and support toward upholding of Ottoman ethnic foods. Recognizing the growing demand for ethnic food, fruity meat dishes of Ottoman cuisine is been brought into businesses.

## Introduction

Traditional dishes reflect the identity and cultural values of the countries [1, 2]. Accordingly, the term heritage food is defined as an element that uses methods, techniques, materials, and utensils in which recipes, stories, cooking methods, and kitchen utensils are transferred from the old generation to the new generation [3]. Traditional dishes play an important role in the formation and development of the food culture and consumption habits of the countries, but also in terms of reflecting the cultural richness [4, 5]. There is increasing awareness of a kind of intangible traditional food heritage that reflects the life of societies. The cultural construction of traditional dishes is becoming increasingly important, with the understanding of the value of culinary skills and the heritage of food and its maintenance [6]. Conservation of this intangible cultural heritage is a protection especially against the processes of globalization and social transformation [7]. The emphasis on culture and gastronomy and the need to protect intangible heritage are similar to the principles of the UNESCO Creative Cities Network. These principles bring together culture and creativity in the development of talents in order to develop and diversify cultural production in the local and global market [8]. In the current era of globalization, the preservation of traditional food heritage and the ability of businesses to maintain traditional dishes; it requires creative and innovative thinking based on valuable resources that the new generation does not have, such as past history and knowledge [9].

Recently, vocational training programs have gained importance in providing a skilled labor force for the food sector [10]. The growth of these sector is directly proportionate to customer satisfaction, and customer satisfaction can be achieved through better service [11]. At this point, professional training is a way to achieve high-quality customer service, professional competence, and increased work performance in an establishment [12]. Professional competence in the culinary field includes a number of qualities such as cultural richness, historical and social knowledge, aesthetics and managerial skills [13].

Cooks are considered to be one of the most crucial elements of human capital necessary for successful service operations [14]. In addition, food is not only representative of countries’ history, culture, and traditions, but it is also a means of interaction between different nations and cultures [15]. Those who are interested in the uniqueness of food in different cultures are enthusiastic about trying different dishes and flavors when they travel to other countries [16]. Also, ethnic foods are increasingly popular in global food markets [17]. This impels kitchen personnel to develop new approaches by using their creativity or combining different concepts in new ways to implement unique methods [18]. The culinary culture of nations is one of the main promotional elements involving unique implications for the local culture [19]. Places that are rich in culinary heritage ought to focus on researching, promoting, and preserving their culinary culture [20]. In recent decades, various ethnic cuisines have gained increasing presence in global food markets [17]. In this framework, it is important to focus on Ottoman culture, which is one of the cumulative constituents of Turkish cuisine. The Ottoman Empire served as home to countless cultures, religions, and this cultural wealth made a positive impact on the culinary culture, creating a unique kitchen [21]. Ethnic meat products are traditionally, culturally, and commercially most valuable food products in different countries throughout the world [22].

It is quite important for nations, ethnic groups, or tribes which went through a long history of agriculture to study their own ethnic foods, develop traditional technologies, introduce and transfer their culture to generations and others [23]. Thus, the aim of this research is to introduce fruity meat dishes in Ottoman cuisine to life in Eskisehir. The sub-goal of this research is to give training to the kitchen staff on both theoretical grounds by introducing them to the culture of Ottoman cuisine and on practical techniques regarding these dishes that they can transfer to next generations.

## Methodology

A mixed research design was implemented in this research. In Table 1, expert opinions were taken on the subject of Ottoman cuisine. The dishes, recipes, and content were determined by interviews with these experts. Document analyses were also performed. As a result of the document analysis and expert opinions, 10 practice sets, each including four dishes, were prepared.

**Table 1:** Information regarding experts

No	Date of birth	Professional experience (years)	Profession	Level of education
1	1941	58	Food writer	Doctorate
2	1956	44	Research associate (retired)	Doctorate
3	1967	32	Professor of Ottoman cuisine history	Doctorate
4	1986	21	Culinary program research associate	Doctorate
5	1976	33	Chef, business manager	Secondary school

To select the participating chefs within the scope of this research, information notices were posted through the electronic advertisements. The announcements were communicated to food establishments in the city of Eskisehir as listed on the Ministry of Culture and Tourism official website [24]. Thus, applications were collected; and due to limitations of training kitchen capacity, the number of instructors, business workloads, manager permits, business cycles, and Covid-19, the number of participating chefs was limited to 12 (Table 2).

**Table 2:** Information about participants

Code	Age	Gender	Professional experience (years)	Type of establishment
P1	53	Male	42	Corporate kitchen
P2	35	Female	15	Corporate kitchen
P3	24	Female	9	Hotel kitchen
P4	28	Male	12	Restaurant kitchen
P5	25	Female	6	Restaurant kitchen
P6	40	Male	21	Hotel kitchen
P7	56	Male	41	Corporate kitchen
P8	42	Male	28	Bistro-pub kitchen
P9	29	Male	14	Hotel kitchen
P10	26	Male	11	Restaurant kitchen
P11	44	Male	29	Culinary education
P12	25	Female	5	Restaurant kitchen

Between August 31 and September 11, 2020, 10 practice training sessions consisting of 40 h of training during weekdays were held with the voluntary participants. The names of the special dishes taught in these courses are given in Table 3.

**Table 3:** Fifteenth–nineteenth century Ottoman fruity meat dishes included in training sets

Day	First dish	Second dish	Third dish	Fourth dish
1	*İstofato Kum Makaronya*	*Kuzu Kızartması*	*Nirbaç*	*Kabuniye*
2	*Levrek Güveç*	*Patlıcan Kayganası*	*Yalancı Balık Dolması*	*Terkibi Çeşidiyye*
3	*Acem Pilavı*	*Kuzu İç Pilav*	*Hünkâr Pilavı*	*Kubuni Pilav*
4	*Kayısı Yahnisi*	*Sarımsaklı Yahni*	*Erikli Yahni*	*Kestane Yahnisi*
5	*Tavuklu Yahni*	*Tatlılı Yahni*	*Sığır Eti Yahnisi*	*Tavşan Yahnisi*
6	*Zerdali Aşı*	*Tuffahiye*	*Mutancana*	*Maydanozlu Köfte*
7	*Terkibi Tuffahiye*	*Patlıcan Dolması*	*Erik Dolması*	*Etli Kavun Dolması*
8	*Hamsi Terbiyesi*	*Mahmudiyye*	*Tatlı Et*	*Curcaniye*
9	*Hulviye-Ferhane Aşı*	*Bamya Bastı*	*Mülebbes Dolma*	*Hısrımiye*
10	*Özbek Pilavı*	*Sikbaç*	*Raşidiye*	*İbrahimiye*

**Fig. 1:**
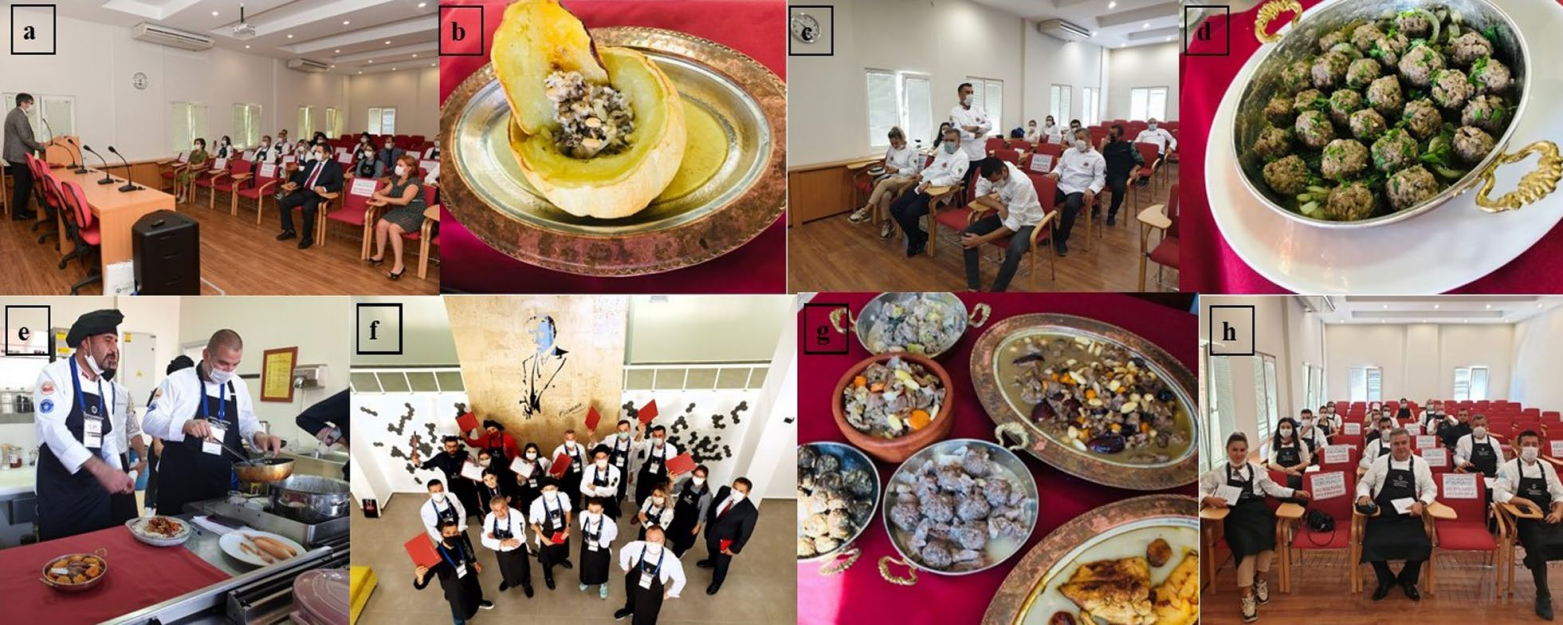
Collage of some photographs from trainings. **a:** Ceremony; **b:** Etli Kavun Dolması; **c:** Information meeting; **d:** Maydanozlu Köfte; **e:** Culinary trainings; **f:** Participants; **g:** Samples of Ottoman fruity meat dishes; **h:** Theoretical training

In the course of the training, the sessions were held in an applied and face-to-face manner (Fig. 1). The training sessions were held for two weeks every weekday for four hours. As a preliminary, training handbooks were distributed, and the participants were informed on codes of conduct and communication and the purpose and scope of the research. The first interview was conducted before the training, and the second interview was held at the end of the last session. Four months after the training, a final case interview was conducted. In this research, qualitative data collection methods such as observation, interviewing, and document analysis were utilized to present the case in a factual and holistic manner [25].

In preparation for the design of semi-structured interview questions (Table 4), a survey of the literature and the opinions of experts who focus on Ottoman cuisine, as listed in Table 1, were utilized. With the permission of the participants, the interviews were recorded, transcribed and analyzed with the content analysis method.

**Table 4:** Some of the questions used in the interviews

Order	Question
1	What is Ottoman cuisine? Can you explain?
2	Can you tell us about the Ottoman dishes you know?
3	Could you give the recipes of fruity meat dishes that you know from Ottoman cuisine?
4	Can you use Ottoman dishes in your business? How?
5	What are the expectations from your customers for Ottoman dishes?
6	What are the opinions of the managers and owners of the business you work for about Ottoman ethnic foods?
7	Do you benefit from ethnic foods in your profession?
8	How do ethnic Ottoman dishes relate to traditions, ancestry, language, history, society, culture, nation, religion or social behavior?

## Results and discussions

**Fig. 2:**
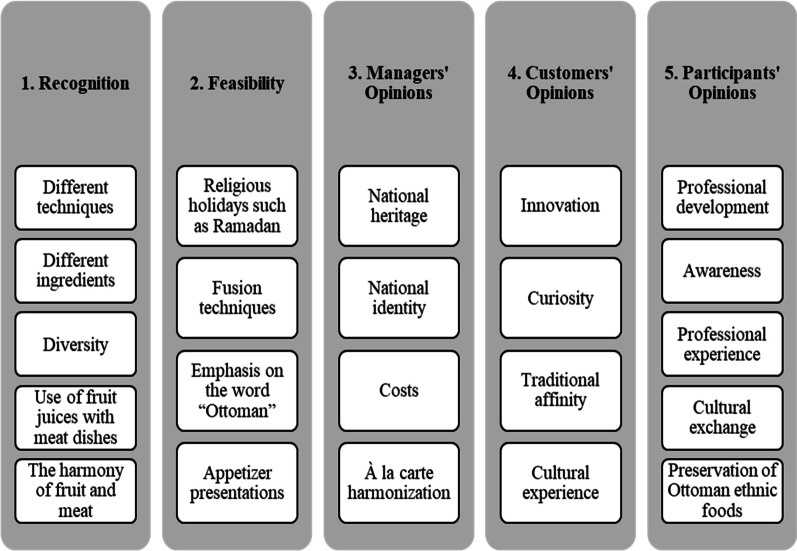
Themes identified in the data analysis

Themes and worthy remarks emerging from data analysis are given in Fig. 2.

### The recognition

The chefs participating in the training remarked that Ottoman cuisine is rich and of great importance to contemporary Turkish cuisine; however, they stated that they do not have extensive knowledge of Ottoman cuisine. They stated that they have limited knowledge of the dishes that belong to the Ottoman cuisine: “This is our past, we hear about Ottoman and Turkish cuisines all the time and are proud of them, yet neither do we utilize them, nor do we know how they actually work” (P4). With such remarks, the chefs expressed that there are many meat dishes they know, yet they are unaware of their original names and ingredients. They stated that this knowledge they will derive from the training will help them improve their professional capabilities. Some participants also mentioned that when they enrolled in this training, they performed some individual research on Ottoman cuisine to satisfy their curiosity. In addition, they voiced their surprise that the diversity of ingredients in these dishes was quite different from contemporary compositions. P12 states that Ottoman cuisine is reminiscent of court kitchen. She mentions that these dishes prepared for Sultans in palace grounds consist of a rich and distinguished variety of dishes from soups to sherbets. However, she has no extensive knowledge of the names, ingredients, and recipes of these dishes. P9 indicated that he does not have enough knowledge about these dishes, since “Ottoman dishes are not too popular these days.” He states that the establishment he is currently employed at offers a large variety of popular dishes from world cuisine but that he is open to new ideas that this training will contribute to his personal skills.

The participants stated that Ottoman cuisine has a great variety of rice dishes and cooking techniques and that stews with various types of meat, and spices were quite common. Additionally, they added that in meat dishes, there was frequent use of various fruits and verjuice from sour grapes, that tomato paste was introduced to Ottoman cuisine in the nineteenth century and that game and poultry were also included in these meat dishes.

The second interviews showed that the participants became more adept at describing and commenting on the Ottoman cuisine and how its various dishes. Consequently, as a result of theoretical and practical training, the participants exhibited changes in their terminal behavior. P5 expressed that in addition to fruity meat dishes included in this training, she also learned additional recipes such as *sirkencübin*, *keşkül-ü fukara*, and *kavun çekirdeği sübyesi.* Also, the participants expressed that they were happy to have the chance to taste, smell, and use many different ingredients that they would not encounter in their current field of work. In this context, they underlined some ingredients such as *musk* derived from the belly fat of a gazelle and genuine saffron, ingredients that are difficult to procure and costly.

In the interviews that took place four months after the training period, the common opinion was that there are many meat dishes in the Ottoman cuisine, and they are difficult to make, but they are quite tasty. P8 highlights the contributions of this training to his career as follows: “Before the training, I could not have guessed how flavors, such as cinnamon, apricots, and grapes, could be mixed into meat dishes with such harmony of taste.” P12 states that “these dishes could well be adapted to contemporary taste with more common substitute ingredients and served in establishments.” In addition, P1 states that “a careful arrangement of okra in the okra stew adds to the visual composition of the dish,” emphasizing the possibility of aesthetically pleasing presentation techniques.

It was determined that chefs require a training period, incentives, and support in order to bring traditional cuisine into food businesses and ensure the preservation of traditional recipes. Recently, an effort can be observed in many countries to redefine their traditional cuisines and culinary cultures as a prominent token of their national identity [26]. These efforts include the preservation of traditional cuisines, the documentation of recipes and cultural knowledge, and working to increase the popularity both within and outside of national borders to formulate a national culinary identity [27–29].

### The feasibility

The chefs participating in this study expressed that they intended to bring fruity meat dishes from Ottoman cuisine to their workplaces, thus reflecting their own cultural identity into their work. Some participants stated that they noted which of the recipes they intended to include during the course of the training period and conferred with their business managers and owners regarding these dishes. Most of the chefs expressed their intention to offer some of these dishes during Ramadan in fast-breaking (*iftar*) meals as small servings. Some chefs employed in meat-oriented businesses stated that they will definitely include some recipes with lamb and will aim to adjust these recipes in accordance with customer feedback and requests. P4 indicated that they will make similar adjustments to their menu. In this context, he remarked that “I am considering including at least a couple of these fruity meat dishes from Ottoman cuisine in our menu. I learned that Ottoman cuisine is very creative with lamb, and I want to implement this into our menu as well.” Similarly, P5 made comments on the new recipes she learned in terms of cost and preparation.

A number of participants maintained that rice recipes in particular could be accommodated in the menus as appetizers. In line with this, P8 made similar comments with other participants regarding the possibility of serving Ottoman dishes on religious holidays:

During Ramadan, I will prepare a menu that is completely comprised of Ottoman dishes for iftar dinners. We conferred with my business owner, and we believe that our customers will be very pleased with experiencing dishes that are endemic to our culture that they have probably been previously unaware of. We understand that these recipes are cultural treasures, and we want to reflect our own culture.

However, because the training period coincided with the Covid-19 pandemic, most kitchens in establishments were temporarily shut down. Hence, it was not possible to evaluate customer feedback to Ottoman fruity meat dishes in the interviews conducted four months after the training period. However, almost all of the participating chefs stated that they are making preliminary preparations parallel with their remarks above and that they have short-term plans to gradually broaden their portfolios as soon as the businesses are once again operating. In this framework, P8, P10, and P12 mentioned that business managers, owners, and other kitchen staff are willing to include traditional cuisine in their menus and that they are eager to implement Ottoman–Turkish dishes in particular. Traditional food preparation techniques can be tokens of cultural identity, can strengthen social identity, and provide ways to interpret culinary methods and national sentiments [30]. Thus, businesses should not only include dishes that customers are familiar with but also a number of traditional dishes. In the food and beverage industry, businesses play an important role in promoting and representing traditional cuisine, improving product and service quality, and providing customers with experiences of national cuisine [31]. Therefore, chefs and food and beverage establishments have the capability to affect society’s approach to traditional cuisines by promoting them and reconstructing their image [32].

### Managers’ opinions

Participants related that their managers at their business places encouraged them to partake in this training course, that they rearranged work shifts in accordance and that their colleagues asked them many questions regarding training. The participating chefs indicated that business managers cared deeply about Ottoman cuisine due its close ties with national identity, they asked them to showcase the skills they acquired during training, they planned to decide on what new items to include on the menu with the help of the chefs and that there will be no problems regarding preliminaries, cooking, and costs. In the meantime, the chefs shared the theoretical information they gained during the training with their colleagues and that when they returned to the kitchens in their establishments, they would also show their colleagues what they learned in practice. P1 accordingly makes the following remarks:

I mentioned fruit dishes to my managers, the vast variety of rice meals in Ottoman cuisine, and different cooking techniques and to be honest they were quite intrigued. We exchanged ideas about the recipes I learned during training and how we can implement them into our menu. They said that they wanted me to make what I have learned; they wanted to taste them. They also believe that it is important to preserve the essence of our identity.

In the interviews held four months after the training period, participating chefs related that their managers believed what they learned from the training could be used in their establishments in time. The interviews showed that some managers were determined to make use of these recipes while others believed that some dishes in certain categories would be quite compatible with Ottoman fruity meat dishes. For instance, they stated that they could utilize fruity meat rice recipes as side dishes to other main course dishes and serve different *stuffed dolma* varieties in small portions as warm starters.

### Customers’ opinions

The participating chefs stated that their guests at their establishments had specifically requested Ottoman food to be included in their menu. Most of the chefs working in hotel businesses remarked that they have world cuisine-oriented menus and that foreign guests in particular expressed their desire to try Ottoman cuisine specialties. Participants employed in restaurants and private establishments, however, stated that they had items from Turkish cuisine in their menu but no Ottoman dishes.

Preference for an ethnic food as a primary dining option or even incorporation of its culinary techniques and ingredients into traditional meals is likely to be highly dependent on the motivation of individual consumers to embrace fusion in diet. Consumers care about their food choice to the extent that their own utility is affected by the attributes of the ethnic food [17]. Additionally, the inclusion of customers in the process of organizing personalized experiences is known to increase customer satisfaction [33]. Therefore, customer feedback and involvement can be an effective means of constructing an establishment’s menu. P4 related that he mentioned the training courses to some of the regular customers, and this aroused their curiosity.

In the final interviews that occurred four months after the training period, due to the pandemic, participants did not have the chance to receive feedback. However, some participants expressed their conviction that in take-away services and feedback from loyal customers, changes in menu will be much welcomed. Thus, making gradual changes in the coming days and having Ottoman fruity meat dishes among these changes will be advantageous to these businesses. Both managers and chefs wanted to carefully evaluate the potential effects of innovative changes in the menu on business performance, customer satisfaction, and customer loyalty. The participants intend to create awareness in customers regarding Ottoman ethnic foods and that they intend to make good use of customer feedback when adding fruity meat dishes to the establishment menus.

### Participants’ opinions

The chefs stated that Turkish cuisine is a synthesis cuisine and that they are excited to include the recipes they learned in this training to their workplaces, both because this cuisine is part of their culture and because customers will be able to experience different flavors. Despite their lack of prior knowledge on Ottoman cuisine, they were able to learn both theoretical and practical aspects of this cuisine. P8, who has 28 years of experience in the sector, expressed that this was the first time he was involved in such a training course and that he was happy to have had this opportunity.

In the interviews conducted four months after the training, the participants claimed that they gained practical skills and cultural knowledge about Ottoman cuisine and that these achievements had a great positive impact on their quality of professional life. Restaurant staff can inform customers of the background of dishes and influence their taste and choices, so it is important to educate chefs in order to raise awareness and interest in traditional dishes and have them attract customers’ attention to these food items [34]. Studies show that the preservation of traditional cuisines is correlated with the training of chefs [35, 36]. When chefs are trained on fruity meat dishes in Ottoman cuisine, they gain practical skills and consciousness of culinary heritage preservation.

## Conclusions

Bringing Ottoman culinary elements to businesses is related to ensuring the continuity of ethnic foods, which are a part of the culture. Since each ethnic food is unique, each individual and culinary professional need to be sensitive toward the unique appeal of each ethnic food [17]. The preliminary interviews showed that participants were not well informed about Ottoman cuisine. The results showed that the participating chefs wanted to be involved in this study to gain vocational skills and knowledge at both theoretical and practical levels. The participants wanted to represent and conserve their own culture and thus considered this training an opportunity to develop their cultural knowledge and professional skills. Business owners asked participating chefs to implement the recipes they learned and inform their colleagues and include them in the menus. The feedback of the participants shows that they want to apply the traditional food heritage as a powerful marketing tool in their businesses. Emphasizing the importance of using the intangible food heritage as a marketing strategy, De-Miguel-Molina et al. carried out to determine the relationship between intangible heritage and gastronomy and to investigate the use of the criteria determined by UNESCO in terms of marketing. They concluded that restaurants should use UNESCO-recognized intangibles as a tool of their marketing strategy for branding purposes. The authors stated that businesses should benefit from anthropological studies on celebration, cultural transmission, cooking style, sensory characteristics, and consumption style in addition to food products [37].

Participants held that fruity meat dishes in Ottoman cuisine were feasible in terms of cost and time consumption, and businesses were willing to implement these dishes. The results show that the participants wanted to learn their thoughts by serving traditional dishes to customers in small portions. They are curious about customers’ willingness to try ethnic dishes and are looking for ways to integrate them into their menus. Kühne et al.’s results showed that consumers and businesses are open to innovations in traditional food products. At the same time, the preservation of the traditional nature of food products has been stated as a necessity for development and innovation in the field of traditional foods [38]. Of the seventeen products tested, products perceived as traditional were preferred as opposed to modernized ones. Current studies show that consumers are willing to try traditional dishes [39]. These results are important; they show that today’s traditional dishes are acceptable and interesting, and they allow businesses to maintain their food heritage in terms of cultural heritage.

As a result of this study, the training was successful in providing participating kitchen staff with vocational skills and knowledge. According to the analysis of the interviews conducted after the training, the following statements can be made: Ottoman cuisine is one of the constituents of national cultural heritage; spices such as black pepper, cinnamon, cardamom, allspice, clove, saffron, and coriander are popular in Ottoman cuisine; there are various types of rice and rice cooking techniques; there are many stews, and most meat products are marinated a day before preparation. In line with the collected data, all the chefs participating in the training reached a level of understanding of Ottoman culinary and skills to implement. Similarly, Mardatillah et al. determined that the main challenge facing businesses is the potential of local resources, availability, and optimization of materials. For this reason, they concluded that knowledge, performance, and talent are important in businesses in order to protect intangible cultural heritage and create brand quality [7]. The results clearly show the importance of restaurant staff's knowledge and practice skills about traditional dishes for the preservation and sustainability of traditional culinary heritage. In addition, the ability of cooks to apply traditional dishes will enable them to transfer their culinary heritage to future generations and create a global image value.

Participants practicing Ottoman cuisine emphasized that it is important to rebuild their traditions in terms of ethnic elements such as nationality, historical background, religion, and social relations. The importance of traditional cuisines as cultural heritages must be acknowledged to preserve and sustain cultural traditions and to compete globally. One way to ensure this acknowledgement is to raise business manager and chef awareness. In this sense, business managers should be approached, and training programs for kitchen staff in local culinary cultures must be organized.

## Data Availability

Not applicable.
